# Methylated-antibody affinity purification to improve proteomic identification of plant RNA polymerase Pol V complex and the interacting proteins

**DOI:** 10.1038/srep42943

**Published:** 2017-02-22

**Authors:** Guochen Qin, Jun Ma, Xiaomei Chen, Zhaoqing Chu, Yi-Min She

**Affiliations:** 1 Shanghai Center for Plant Stress Biology, Shanghai Institutes for Biological Sciences, Chinese Academy of Sciences, 3888 Chenhua Road, Shanghai 201602, P. R. China; 2 Shanghai Chenshan Plant Science Research Center and Shanghai Chenshan Botanic Garden, Shanghai Institutes for Biological Sciences, Chinese Academy of Sciences, 3888 Chenhua Road, Shanghai 201602, P. R. China; 3 Centre for Biologics Evaluation, Biologics and Genetic Therapies Directorate, Health Canada, Ottawa, Ontario, K1A 0K9, Canada

## Abstract

Affinity purification followed by enzymatic digestion and mass spectrometry has been widely utilized for the sensitive detection of interacting proteins and protein complexes in various organisms. In plants, the method is technically challenging due to the low abundance proteins, non-specific binding and difficulties of eluting interacting proteins from antibody beads. In this report, we describe a strategy to modify antibodies by reductive methylation of lysines without affecting their binding properties, followed by on-bead digestion of bound proteins with endoproteinase Lys-C. By this method, the antibody remains intact and does not interfere with the downstream identification of interacting proteins. Non-specific binding proteins were excluded using ^14^N/^15^N-metabolic labeling of wild-type and the transgenic plant counterparts. The method was employed to identify 12 co-immunoprecipitated protein subunits in Pol V complex and to discover 17 potential interacting protein targets in *Arabidopsis*. Our results demonstrated that the modification of antibodies by reductive dimethylation can improve the reliability and sensitivity of identifying low-abundance proteins through on-bead digestion and mass spectrometry. We also show that coupling this technique with chemical crosslinking enables in-depth characterization of endogenous protein complexes and the protein-protein interaction networks including mapping the surface topology and post-translational modifications of interacting proteins.

Protein-protein interactions play an essential role in maintaining cellular protein function in various biological processes. A common proteomics method to study protein-protein interactions and the assembly of protein complexes utilizes antibody-based affinity purification of protein targets and the interacting partners followed by mass spectrometric identification and quantification^1^,^2^,^3^. Using this protocol, the antibody bound intact proteins are always eluted using either a denaturing buffer at low pH (*e.g.* glycine-HCl), or sodium dodecyl sulfate (SDS) detergent solution and SDS-PAGE separation. However, the detection of endogenous plant interacting proteins using enzymatic digestion of a protein mixture and subsequent analyses with liquid chromatography coupled with tandem mass spectrometry (LC MS/MS) can be technically challenging due to the low abundance of proteins of interest and the interference from relatively high abundance contaminants such as immunoglobulin G (IgG) and chlorophyll proteins. Alternatively, purified protein complexes can be cleaved by on-bead digestions, and the resulting peptides are then eluted from antibody beads and analyzed by LC MS/MS. This facilitates the identification of low abundance target proteins and those that are difficult to unbind from the antibody on which they are typically immobilized. However, the approach results in the generation of high abundance antibody peptides, which contaminate samples and hinder the identification of proteins of interest. It is therefore beneficial to develop a technique, e.g. utilizing chemical modifications, to block enzymatic cleavage sites on the primary antibodies to prevent their digestions. Here we describe such a method to allow the sensitive detection of low-abundant interacting proteins and the comprehensive characterization of protein complexes in plants.

Previous studies showed that the reductive dimethylation of active lysine residues to generate stable products at mild reaction conditions does not change the intrinsic charge on a protein, and possesses little or no detectable effect on biochemical properties of the protein structure^4^,^5^,^6^. One application that has benefitted proteomics research is the utility of reductively methylated trypsin, which increases the stability of this endoproteinase by reducing its susceptibility to autolysis. The modified trypsin has been widely used for proteomic analyses by LC MS/MS^6^. Reductive methylation has also been considered as a simple, inexpensive and highly efficient technique for improving the quality of protein crystals for 3D protein structural studies^7^,^8^,^9^,^10^. With the high labeling efficiency and selectivity of the modification at lysine residues, the reductive dimethylation of intact proteins or proteolytic peptides enabled reliable and accurate quantification of proteins^11^. Using a similar strategy, we have established an improved on-bead digestion protocol that utilizes dimethylated mouse monoclonal antibodies to pull down the interacting proteins of the RNA-silencing enzyme Pol V complex. The modified lysine residues at the surface of the antibodies were resistant to the protease cleavage, and thus binding protein targets and interacting partners could be digested on-bead by endoproteinase Lys-C. After removal of antibody beads, the resulting peptides were subsequently extracted and further digested by a second enzyme such as trypsin to yield sufficient peptide fragments to eventually achieve high sequence coverage of proteins for confident protein identification and quantification.

Pol V is a plant-specific nuclear RNA polymerase that plays a critical role in RNA-directed DNA methylation (RdDM) and short-interfering RNAs (siRNA)-mediated transcriptional gene silencing^12^. The Pol V complex consists of 12 nuclear RNA polymerase E (NRPE) subunits. The large subunit, NRPE 1 (Gene AT2G40030 encoding 1976 amino acids), binds to NRPE 2 to form the catalytic core structure, and several low-abundance subunits of NRPE 3a/3b, NRPE 10, NRPE 11 and NRPE 12 are involved in the complex Pol V assembly^12^,^13^. Therefore, the study of protein interactions and post-translational modifications among individual components as well as the interacting partners of Pol V complex will increase our understanding of the protein functions in RdDM mechanisms and their involvements in transcriptional gene silencing (TGS) or post-transcriptional gene silencing (PTGS) stimulated by biotic or abiotic stresses^14^.

## Results and Discussion

### Highly sensitive detection of the protein subunit compositions of Pol V complex using dimethylated antibodies

**Figure 1:**
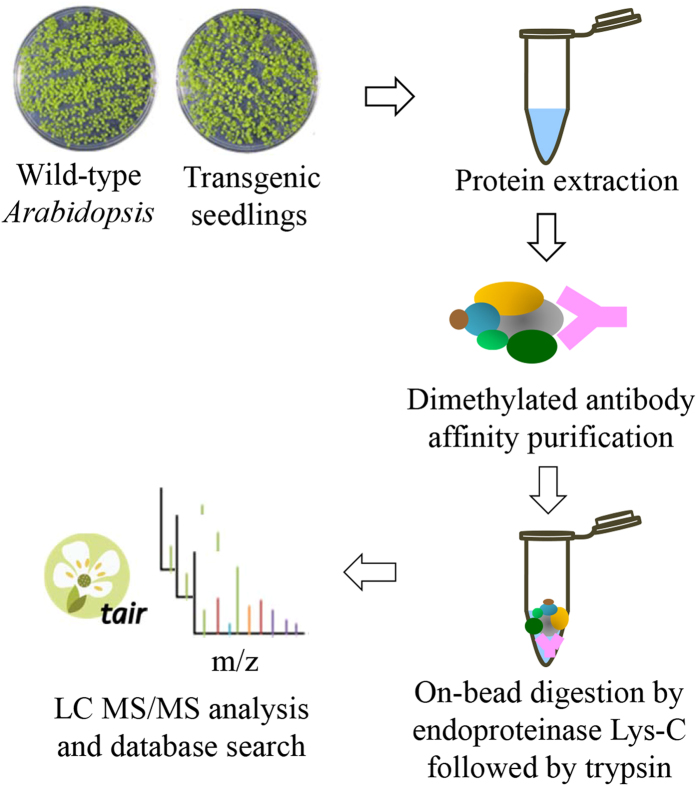
The analytical workflow of protein complexes by dimethylated antibody purification and mass spectrometry. Proteins were extracted from *Arabidopsis* seedlings of wild-type and transgenic plants and then subjected to affinity purification using dimethylated antibody. After on-bead digestion by endoproteinase Lys-C followed by trypsin, the resulting peptides were identified by Orbitrap LC MS/MS.

A well-established protocol described previously^10^ was used for reductive dimethylation of lysine residues on the surface of antibody beads at near physiological conditions (pH 7.5). Amine-borane complexes were selected as the reducing reagents because of their solubility and stability in aqueous solution. After removal of the unreacted formaldehyde and the excess reducing reagent, the formed N,N-dimethyllysine residues of antibodies were resistant to enzymatic digestions by preferred enzymes of trypsin or endoproteinase Lys-C. The experimental workflow for determining interacting proteins by immunoaffinity purification with dimethylated antibody followed by on-bead digestion and LC MS/MS analyses is illustrated in Fig. 1. To explore the utility of this protocol, we purified the Pol V complex from 14-day NRPE1-FLAG transgenic *Arabidopsis* seedlings to evaluate the binding affinity and efficacy of the modified antibodies. Each experiment was performed on three sets of biological samples, and the affinity pull-down proteins identified using dimethylated antibodies were compared to those purified by non-methylated antibodies. The control experiments were carried out using non-methylated anti-FLAG antibody purification followed by either on-bead trypsin digestion alone (control 1), or on-bead digestions with proteinase Lys-C followed by trypsin digestion (control 2). All 12 subunits of Pol V complex were identified in the experiments, whether the antibodies were dimethylated or unmodified. This suggested the dimethylation of the NRPE1-FLAG antibodies does not decrease their binding capacity by a large degree. Database searching on the LC MS/MS datasets identified dimethylation at 61% of the lysine residues on the mouse anti-FLAG antibody, which was expected to be modified predominantly on its surface region. Similar results were obtained by the affinity purification and mass spectrometric analyses of the on-bead enzymatic protein digests purified by other antibodies such as anti-HA (data not shown). The reductively dimethylated antibodies were able to maintain a high binding affinity to the tagged proteins and interacting partners, and the chemical modification by lysine methylation was found to have no effect on the antibody surface structure and antigenic sites.

**Figure 2:**
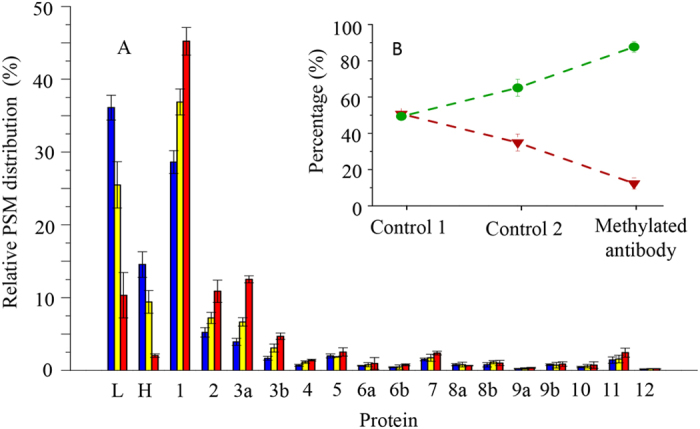
Relative peptide-spectrum-match (PSM) counts of antibodies and the protein subunits of Pol V complex. The peptides were generated from the immunoprecipitated proteins (“Control 1”: non-methylated antibody, on-bead trypsin digestion; “Control 2”: non-methylated antibody, on-bead digestion with endoproteinase Lys-C and then cleavage by trypsin; “Methylated antibodies”: antibodies modified by dimethylation at lysine residues, on-bead digestion with endoproteinase Lys-C and then cleavage by trypsin). Error bars are standard deviations from triplicate experiments. (**A**) Relative PSM counts from individual Pol V complex proteins as well as antibody light (L) and heavy (H) chains. Blue, yellow and red columns represent results from “Control 1”, “Control 2” and “Methylated antibody” purification, respectively. Protein identification and PSMs were generated by Mascot database search against TAIR10. (**B**) The insert is shown the total PSM distribution of antibodies (purple triangles) versus the Pol V complex (green circles), and the dashed line is shown the tendency of the PSM changes.

Semi-quantitative LC MS/MS analyses of the protein digests from three sets of experiments showed that the amount of antibody fragments in the purified Pol V complex has reduced from 50.65% of the non-methylated antibodies (Control 1) to 12.33% of the dimethylated antibodies based on peptide-spectrum-match (PSM) counts (Fig. 2, Supplementary Information Table S1). Correspondingly, the total PSM counts from 12 protein subunits in the Pol V complex increased from 49.35% to 87.67% when the dimethylated antibodies were utilized to replace non-methylated antibodies for protein purification. As shown in Fig. 2, the relative distribution of individual proteins from affinity pull-down Pol V complex by endoproteinase Lys-C digestion plus trypsin has higher PSM distribution (coloured in yellow) than that eluted by on-bead digestion with trypsin only (coloured in blue). The results agree that the additional digestion by endoproteinase Lys-C followed by trypsin typically increase the protein sequence coverage obtained by LC MS/MS analyses. In contrast, the relative distributions of antibody chains have yielded an opposite consequence by such digestions. The high-intensity antibody fragments in the experiment of “Control 1” was caused by the on-bead tryptic digestion where the large amount of antibodies were available. In the cases of the “Control 2” and “Methylated antibody” purification, the less-contaminated antibody fragments in the samples were resulted from on-bead digestion with endoproteinase Lys-C followed by trypsin cleavage on the eluted peptides, which might be due to the difficult elution of large antibody peptides from the endoproteinase Lys-C digest on the pre-crosslinked antibody beads of which were immobilized (“Control 2”) and the un-cleaved antibody sites of dimethylated lysine residues (“Methylated antibody” purification). Enhancement of the MS detection sensitivity of this method has also reflected on the use of the low amount of starting materials at 3 g of *Arabidopsis* seedlings, whereas an earlier reported method required 150 g of *Arabidopsis* callus or leaf tissue^12^. In the initial experiments, we thus demonstrated that the proteomic method combining reductively methylated antibody affinity purification with on-bead digestion can dramatically reduce the interference of antibody fragments and boost the detection sensitivity of subunit compositions of a protein complex.

### Interface mapping of the protein-protein interactions of Pol V complex

To map the protein interface of intermolecular interactions of the Pol V complex, we used chemical cross-linking and mass spectrometry to identify the cross-linked peptides of the Pol V subunits and the closely interacting proteins. Water-soluble bis (sulfosuccinimidylsuberate) (BS^3^) was selected as the crosslinking reagent, which contains an amine-reactive N-hydroxysuccinimide (NHS) ester at both ends to react with primary amines such as the side chain of lysine residues and the N-terminus of a protein. Because most lysines on the antibody surface were protected by dimethylation, on-bead crosslinking predominantly occurred between the purified Pol V protein subunits and the interacting partners. After enzymatic cleavage of proteins, the formation of inter-crosslinked peptides resulted in a molecular mass increase of 138.0681 Da caused by incorporating the crosslinked group of -C(O)CH_2_CH_2_CH_2_CH_2_CH_2_CH_2_C(O)- between two peptides. Online database search using the Batch-Tag Web (ProteinProspector, University of California, San Francisco)^15^ identified 20 inter-crosslinked peptides as listed in Supplementary Information Table S2. Overall, the results revealed topological locations of the interacting protein subunits between NRPE1 and NRPE2, NRPE1 and NRPE3, NRPE1 and NRPE5, NRPE1 and NRPE6, NRPE2 and NRPE3, NRPE2 and NRPE4 in the Pol V complex, which were consistent with the interaction model of the protein subunits based on the yeast Pol II crystal structure as reported previously^12^.

**Figure 3:**
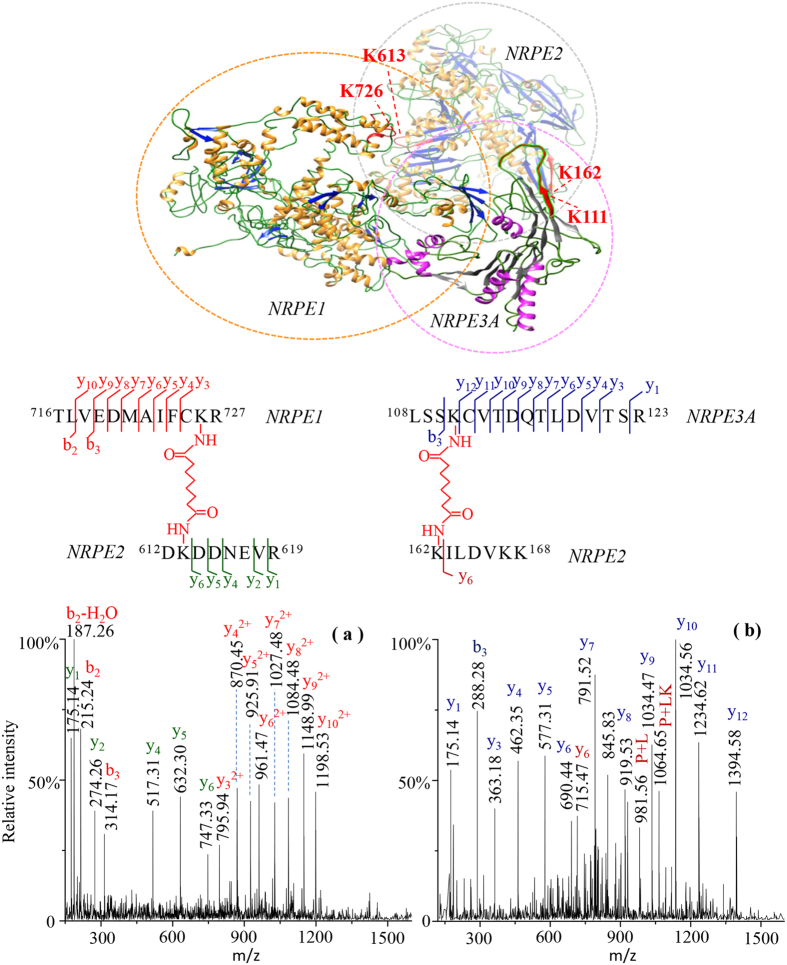
Structural interface mapping of protein subunits in Pol V complex. (**a**) MS/MS spectrum of the inter-crosslinked peptide NRPE1-NRPE2 of triply charged ion at m/z 870.757. Fragment peaks of the C-terminal y_n_ ions and the N-terminal b_n_ ions are annotated in red for NRPE1 and green for NRPE2. (**b**) MS/MS spectrum of the inter-crosslinked peptide NRPE2-NRPE3A of triply charged ion at m/z 930.830. Fragment peaks of the C-terminal y_n_ ions and the N-terminal b_n_ ions are annotated in brown for NRPE2 and dark blue for NRPE3A. The fragments of P + L and P + LK are labeled as the cleavage product ions of the specifically modified lysines derived from BS^3^ cross-linking reagent and tetrahydropyridine cross-linked peptide^15^. The topological localizations between two interacting peptides were highlighted in red as illustrated on the top panel of the structure modeling of *Arabidopsis* NRPE1, NRPE2 and NRPE3A by Phyre2 program, using the known 3D structures of the protein homologues of the DNA-directed RNA polymerase II subunits rpb1 from schizosaccharomyces pombe (PDB code 3H0G), rpb2 (PDB code 5FLM), and RNA polymerase III (PDB code 5FJ8) as templates with 100% confidence.

Analyses of the high scoring peptides of the interacting proteins retrieved by Batch-Tag database search provided a broad view of the assembly of inter-crosslinked subunits of the large Pol V complex such as NRPE1, NRPE2 and NRPE3. Figure 3a shows the MS/MS fragmentation of a crosslinked peptide at the triply charged ion at m/z 870.757 comprising residues 716–727 of NRPE1 and residues 612–619 of NRPE2. This peptide yielded a set of C-terminal y_n_ ions matching the unmodified y_1_-y_6_ product ions of the NRPE2 peptide as well as the BS^3^ crosslink-modified y_3_-y_10_ product ions of the NRPE1 peptide. We were therefore able to identify a cross-linkage between Lys-726 of NRPE1 and Lys-613 of NRPE2. Similarly, the cross-linked sites of Lys162 at NRPE2 and Lys111at NRPE3A were reliably identified in the MS/MS pattern of the triply charged ion at m/z 930.830 as shown in Fig. 3b. Structure modeling of the full-length sequence of NRPE1 (AT2G40030, 1976 amino acids) using Phyre2 retrieved a protein homologue of DNA-directed RNA polymerase II subunit rpb1 from *schizosaccharomyces pombe* (PDB code 3H0G), which shares 59% amino acid identities^16^. The N-terminal sequence of NRPE1 at residues 7–1237 was modeled with 100% confidence using the template. Likewise, two predicted structures of NRPE2 (AT3G23780, 1172 amino acids) and NRPE3A (AT2G15430, 319 amino acids) was modeled at the sequence region of NRPE2 residues 25–1167 and NRPE3A residues 2–299, respectively, based on the homologues of DNA-directed RNA polymerase II subunit rpb2 (PDB code 5FLM) and RNA polymerase III (PDB code 5FJ8) with 100% confidence. As such, a part of the structure modeling of Pol V complex could be built by the three inter-crosslinked peptides of adjacent protein subunits NRPE1-NRPE2, NRPE2-NRPE3A, and NRPE1-NRPE3A (Supplementary Information Table S2) as shown at the upper panel of Fig. 3, suggesting the dimethylated antibody purification followed by chemical crosslinking on beads provides a highly sensitive method to map the interface of Pol V complex assembly.

### Quantification of interacting proteins with NRPE1

**Figure 4:**
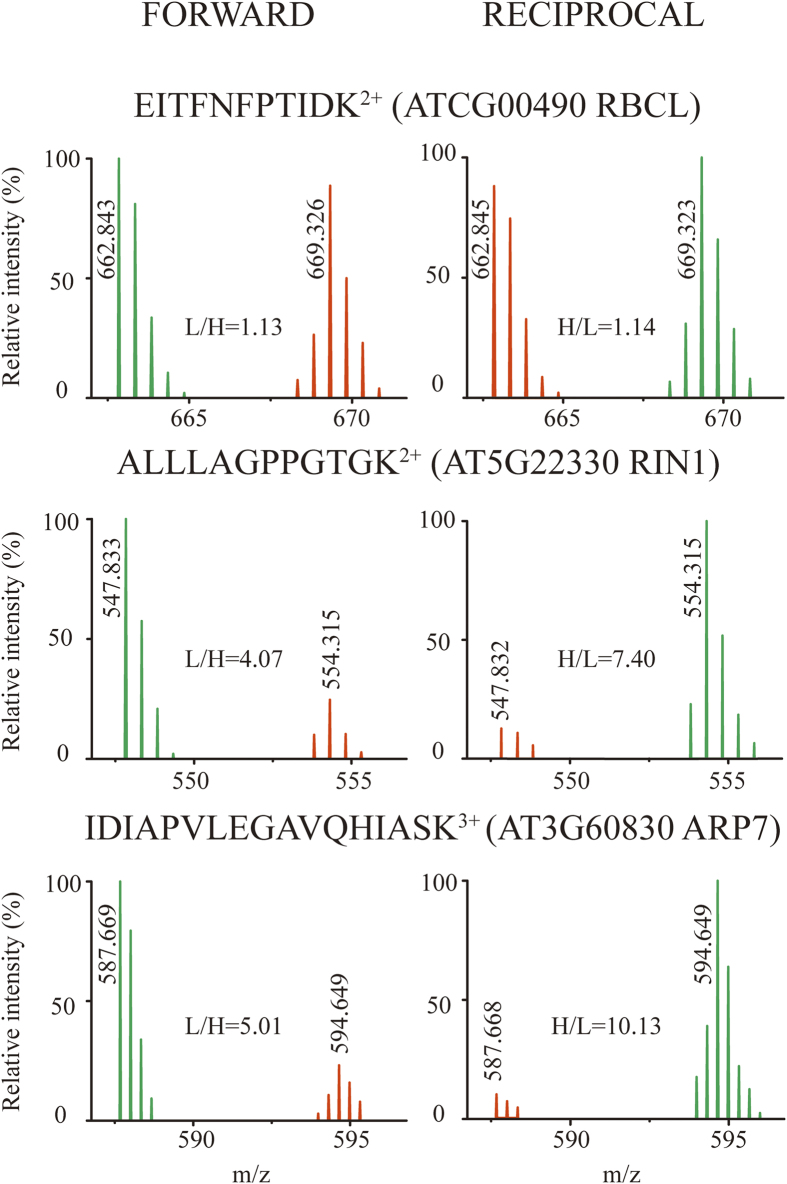
Accurate protein quantification of interacting protein targets with Pol V complex by forward and reciprocal ^15^N-metabolic labeling and LC MS/MS analyses. In the “FORWARD” experiment, the ^14^N-isotope was labeled on the NRPE1-FLAG transgenic plants (peaks in green), and ^15^N-isotope was labeled on wild-type Arabidopsis (Col-0) (peaks in orange). In the “RECIPROCAL” experiment, the reverse isotope labeling was the case. The ratios of H/L (or L/H) of three representative proteins were shown to have unchanged intensities (H/L = ~1) on the non-specific binding protein of ribulose bisphosphate carboxylase large subunit (RBCL). However, significant changes in abundance were observed for antibody-specific binding proteins of RuvB-like protein 1 (RIN1) and actin-related protein 7 (ARP7).

Protein quantification by stable isotope ^15^N-metabolic labeling was employed to distinguish the interacting proteins from non-specific binding partners on antibody beads. Accurate measurements were achieved by the forward and reciprocal ^15^N-siotope labeling experiments in parallel. Protein abundances of *Arabidopsis* seedlings were measured by LC MS/MS analyses through a comparison of two sets of biological materials on Col-0 wild-type (light ^14^N-isotope labeled proteins, L) and NRPE1-FLAG transgenic seeds (heavy ^15^N-isotope labeled proteins, H) at the same growth conditions, and *vice versa* in the reciprocal replicates (Fig. 4, Supplementary Information Figure S1). Similar H/L ratios of the relative protein abundances were obtained in the two experiments. The overall expression levels of rubisco activase, ribulose bisphosphate and other non-specific proteins showed no differences as measured (H/L ratios at ~1), and the 12 NRPE subunits of Pol V complex were merely appeared in the dimethylated antibody pull-down fraction of NRPE1-FLAG transgenic seedlings rather than the wild-type materials. In contrast, a value increase of over 1.5 folds was observed in the abundances of proteins such as glycine rich protein 7 (GRP7), RuvB-like protein 1 (RIN1), DNAJ homologue 3 (J3), PYK10-binding protein 1 (PBP1), 2,3-biphosphoglycerate-independent phosphoglycerate mutase 2 (IPGAM2), argonaute 4 (AGO4), RNA-directed DNA methylation 4 (RDM4), actin-related protein 7 (ARP7), RuvB-like helicase, OB-fold-like protein, heat shock protein 70 (HSP70), regulatory particle AAA-ATPase 2B (PRT2B) and two unknown proteins (AT1G13930 and AT3G44690). The H/L ratios for these proteins ranged from 3.32 to 14.53 and they were therefore considered as potential interacting partners of Pol V complex (Supplementary Information, Table S3).

These data support previous reports that the known nuclear proteins of *Arabidopsis* AGO4 and RDM4 were physically interacted with Pol V transcripts affecting functions in epigenetic regulation and plant development^17^,^18^,^19^. Our results using relative protein quantification showed additional interacting protein candidates of RIN1 and RuvB-like helicase which are in good agreement with LC MS/MS analysis and subsequent Batch-Tag search mapping of interacted peptide pairs between the subunits of Pol V complex and those interacting proteins (Supplementary Information Table S2). ARP7 and RIN1 are essential components of chromatin remodeling to assemble a wide variety of multi-protein complexes that are involved in the regulation of chromatin structure, transcription regulation, and DNA repair^20^,^21^. HSP70 is a chaperone protein that plays an important role in protein-protein interactions by stabilizing interacting proteins to ensure correct folding and proper functions. Analyses by Gene Ontology (GO) annotation indicated these interacting protein candidates all have the same subcellular localization at the nucleus as that of Pol V complex. Consequently, the collective data suggest there are a number of additional proteins interacting with the Pol V complex, although these putative targets need further biochemical validation such as Co-IP pull down, subcellular location, gene overexpression and knockout experiments.

### Dynamic interaction with multiple protein kinases of NPRE1

The dynamic interactions between kinases, phosphatases and protein substrates are weak and transient. This renders it difficult to detect the direct interacting partners and complex signaling networks involved in cellular regulatory processes by affinity purification and mass spectrometry. To overcome this problem, MS-based quantitative phosphoproteomics using plant materials with either kinase knockouts or overexpression phenotypes has been adopted. The approach facilitates the identification of potential interacting protein targets, but faces the technical challenges due to the need for time-consuming analyses of large-scale proteomics data sets and the complexity resulting from indirect protein interactions. Improved sensitivity of dimethylated antibody purification in LC MS/MS detection of protein subunits in Pol V complex enabled in-depth analyses of post-translational modifications of the endogenous plant proteins, and allowed the discovery of dynamic interactions of kinases and ubiquitin ligases through specifically recognized protein substrates and the conserved structure binding motifs. Using on-bead digestions and LC MS/MS analyses of the purified Pol V complex, we have reliably identified 6 phosphorylated peptides and 2 ubiquitinated peptides in NRPE1 based on high Mascot searching scores and accurate mass measurements (Table 1). The detailed structural elucidations of phosphopeptide sequences were illustrated in the MS/MS spectra in which were observed the intense series of C-terminal y_n_ fragment ions (Supplementary Information Figure S2). Two unique peptides of ^1293^FED**S**ADFQNLHDEGKPSGANWEK^1315^ and ^1372^SD**S**GGAWGIK^1381^ were phosphorylated at residues Ser1296 and Ser1374, which bear the specific sequence motifs of SxD and SD, suggesting the possible NPRE1 interaction with sucrose nonfermenting 1 (SNF1)-related protein kinase 2 (SnRK2s)^22^. Another phosphopeptide at the sequence region of ^1384^DADADT**T**PNWET**S**PAPK^1400^ contained two monophosphorylation sites which localize at the residues of either Thr1390 or Ser1396 followed by proline (S/T-P motifs), indicating a mitogen-activated protein kinase (MAPK) might have been involved in the proline-directed phosphorylation signaling of NRPE1 to regulate the NRPE1 protein function^23^. In addition, two phosphorylation sites at the peptides of ^1568^KN**S**ETELGPAAMGNWDK^1584^ and ^1729^LDSFTSEEQELL**S**DVEPVMR^1748^ were identified at residues Ser1570 and Ser1741, which were localized at the conserved motifs of SExE and SxxE, indicating the possible interaction of NRPE1 with calcium-dependent protein kinase (CDPK) and SNF1-related kinase 1 (SnRK1)^23^. Indeed, those kinases including CDPK2, CDPK3, CDPK6, CDPK9 and SnRK2.1 have been identified by LC MS/MS analyses of the immunoprecipitation eluates of the NRPE1-FLAG transgenic plants by the dimethylated antibody purification method (data are available *via* ProteomeXchange with identifier PXD005422).

MS/MS analyses revealed that two highly conserved peptides at the protein sequence regions of residues 1453–1467 and 1517–1533, sharing amino acids identity of 71% by BLAST similarity search, were mono-ubiquitinated at residues Lys1453 and Lys1517 (Supplementary Information Figure S3). The ubiquitin-interacting motif (UIM) was established to have sextet arrays of conserved sequence KKN(S/K)S(/I)ETE(/D) in the region of residues 1451–1573 which were likely interacted with ubiquitin through specific ubiquitin-ligases (E2/E3) in plants. No structural homologue was found at the C-terminal sequence of residues 1238–1976 by PSI-Blast search and Phyre2. Mass spectrometric analyses thus provided evidence for a previously unrecognized regulatory structure domain at residues 1293–1573 of NRPE1, where the post-translational phosphorylation and mono-ubiquitination were inferred to regulate protein functions through dynamic protein-protein interactions with kinase, phosphatase, ubiquitin-ligase and de-ubiquitinating enzyme.

**Figure 5:**
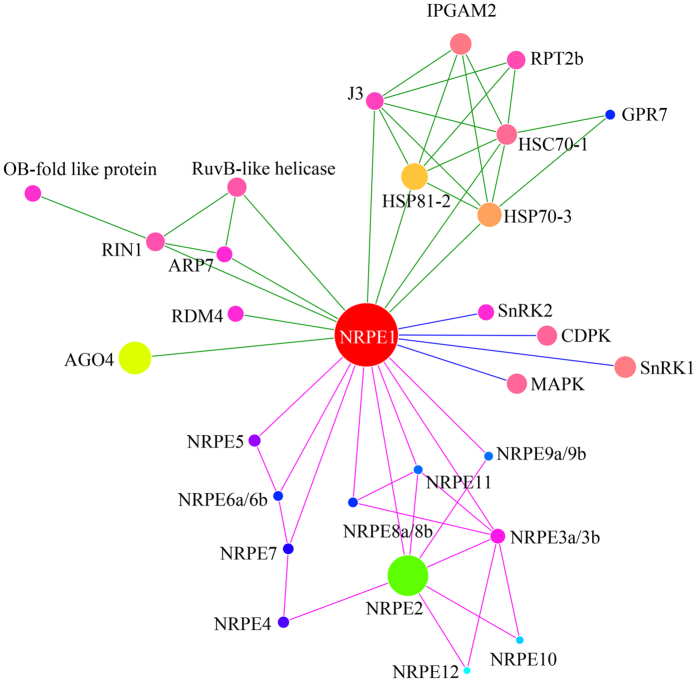
Predicted protein interaction network of Pol V complex as determined by LC MS/MS analyses. The interacting protein candidates were analyzed by the STRING database, and then loaded into the Cytoscape program to visualize protein interaction pathways. Relative sizes of the balls are drawn to be consistent with the predicted masses of the protein sequences.

In conclusion, the use of dimethylated antibody affinity purification, chemical crosslinking, ^15^N-metabolic labeling, on-bead digestion and LC MS/MS mass spectrometry has been demonstrated to be a highly sensitive method for the determination of Pol V complex and the interacting protein targets. Dimethylation of antibodies generated the modified lysine residues which were resistant to the enzymatic cleavage by endoproteinase Lys-C, and thus reduced the detection of interfering antibody fragments in the subsequent LC MS/MS analyses. High efficiency of chemical crosslinking was achieved selectively on the enriched protein complexes and interacting proteins, rather than dimethylated antibodies, to identify the interface of protein-protein interaction domains. The technique was demonstrated to be capable of identifying low abundance protein subunit compositions, intermolecular cross-linkage peptides and a wide range of protein post-translational modifications. As a result, we have identified all 12 protein subunits of Pol V complex and 17 interacting protein targets, and gained insights into the structure assembly of Pol V complex. We have also established its protein-protein interaction network (Fig. 5) based on the putative protein interactions with kinases (MAPKs, CDPKs, SnRKs) and ubiquitin-ligases, and identified a previously unrecognized C-terminal regulatory domain by proteomic analyses of the post-translationally modified phosphorylation and mono-ubiquitination, as well as the kinase-substrate recognition motifs of the protein subunit NRPE1. The methodology described here therefore provides a general tool for large-scale analysis of endogenous protein complexes and associated interacting proteins in plants and other organisms.

## Materials and Methods

### Plant materials

Seeds of *Arabidopsis thaliana* ecotypes *Columbia-0 (Col-0*) and NRPE1-FLAG transgenic plants were surface-sterilized by 0.5% sodium hypochlorite solution, and then mixed with 0.1% (w/v) agar solution and sown on the agar growth media containing half-strength Murashige & Skoog (1/2 MS) medium, 1% (w/v) sucrose and 0.7% (w/v) agar at pH 5.7. After incubation in the dark for 2 days at 4 °C, the seedlings were then transferred to the plant growth chamber at 22 °C under long-day conditions (16 h light, 8 h dark) for 14 days. The materials were collected and immediately frozen in liquid nitrogen and stored at −80 °C until use.

### *In vivo*^15^N-isotope metabolic labeling of proteins in Arabidopsis

Metabolic labeling of proteins in wild-type *Arabidopsis* (Col-0) and NRPE1-FLAG transgenic plants was performed using the light (^14^N) and heavy nitrogen (^15^N) stable isotopes, respectively, as described previously^24^,^25^. In the ^15^N-incorporated medium, the heavy labeling reagents of ^15^NH_4_^15^NO_3_ and K^15^NO_3_ (Cambridge Isotope Laboratories, Andover, MA) were used to replace those containing ^14^N-isotope.

### Dimethylation of antibody beads

Anti-FLAG M2 affinity gels (Sigma, St. Louis) were chemically modified by dimethylation according to the protocol reported previously^10^. Briefly, 3 μl of 1 M amine-borane complexes (Sigma, St. Louis) and 6 μl of 1 M formaldehyde were added to 50 μl of suspended antibody beads in 100 μl of ice-cold 50 mM HEPES buffer (pH 7.5) containing 150 mM NaCl. The steps were repeated twice for 20 minutes. After reductive methylation, the antibody beads were washed with the HEPES buffer for three times and immediately used for affinity purification of proteins.

### Affinity purification and on-bead digestion

Approximately 3 g of seedling were ground to fine powder with a mortar and pestle in liquid nitrogen and suspended in 9 ml of lysis buffer (50 mM Tris-HCl, pH 7.5, 150 mM NaCl, 5 mM MgCl_2_, 10% glycerol, 0.1% NP-40, 0.5 mM DTT, 1 mM PMSF, and protease inhibitor cocktails). The lysate was centrifuged for 15 min at 14,000 g, and 50 μl of anti-Flag antibody beads were added to the supernatant. After incubation at 4 °C with rotation for 3 h, the beads were washed with 2 ml of lysis buffer for four times and then twice with 1 ml of 50 mM NH_4_HCO_3_. The bound proteins were digested on-bead by the addition of 100 μl of 100 mM NH_4_HCO_3_ containing 1 μg of Lys-C (Promega, Madison, WI) overnight at 37 °C. The crosslinked anti-FLAG antibody beads were then removed by centrifugation. To increase the number of detected peptides by LC MS/MS analysis and the subsequent protein sequence coverage through database search, the supernatant containing large proteolytic peptides were further digested with 2 μg of sequencing-grade trypsin (Promega, Madison, WI) in 50 mM NH_4_HCO_3_ for 7 h at 37 °C. All peptides were purified using StageTips prior to LC MS/MS analysis. The concentration of peptides was determined using the BCA assay.

### On-bead chemical cross-linking following affinity purification

Following dimethylated antibody affinity purification, the beads were washed two times with 1 M NaCl lysis buffer, and then twice with 30 mM HEPES buffer (pH 7.5) containing 150 mM NaCl. The structure of the immunoprecipitated Pol V complex was kept stable by maintaining physiological conditions in the same buffer solution through the chemical cross-linking reaction, which was performed on the resuspended beads in 30 mM HEPES buffer (pH 7.5) containing 150 mM NaCl and 5 mM BS^3^ (Sulfo-DSS, Thermo Scientific) for 1 h at room temperature in the dark. The reaction was quenched by removing HEPES buffer followed by centrifugation. The cross-linked proteins were eluted and reduced from beads by the elution buffer (4% SDS, 100 mM Tris/HCl, 0.1 M DTT, pH 7.6) at 95 °C for 5 min, and then alkylated with iodoacetamide and digested by trypsin using the filter-aided sample preparation (FASP) method on a centrifugal filter unit (10 kDa MWCO). The resulting peptides were acidified with 0.1% formic acid (FA) before mass spectrometric analysis.

### LC MS/MS analysis of the enzymatic digests

Peptides were reconstituted in 0.1% FA and subsequently analyzed by nanoAcquity ultra performance LC (Waters, Milford, MA) and Orbitrap Fusion mass spectrometry (Thermo Fisher Scientific, Watham, MA). The peptides were trapped by a 2G-V/MT Trap symmetry C18 column (5 μm particles, 180 μm ID × 20 mm length) at the flow rate of 3 μl min^−1^ for 5 min, and separated on a BEH130 C18 analytical column (1.7 μm particles, 100 μm ID × 100 mm length) at 350 nl min^−1^. Peptides were eluted using mobile phases consisting of solvent A (0.1% FA) and solvent B (ACN/0.1% FA) through a linear gradient from 8% to 35%, and then 50% of solvent B at a duration of 95 min. Data-dependent MS/MS acquisition was performed following a full MS survey scan by Orbitrap at a resolution of 60 000 over the m/z range of 350–1800, and MS/MS measurements of the top 20 most intense precursor ions by HCD scans. The target values of automatic gain controls (AGC) were set up to 200,000 for Orbitrap MS and 10,000 for ion-trap MS/MS detection. Dynamic exclusion was enabled for 60 s.

### Protein identification and quantification

The MS raw data from the LC-MS/MS analyses were converted to MGF files using the Proteome Discoverer 1.4 software (Thermo Fisher Scientific, Watham, MA) and then searched against the database of The *Arabidopsis* Information Resource (TAIR10; downloaded on 6 January 2014; 35 386 sequences) using Mascot Daemon 2.5 (Matrix Science, London, UK). The search parameter for tryptic digestion was restricted to two missed cleavages of proteins. Deamidation of Asn or Gln, and oxidation of Met were considered as variable modifications. Mass tolerances were set up to 10 ppm for Orbitrap MS ions, and 0.8 Da for ion-trap MS/MS fragments. Peptide assignments were filtered by an ion score cut off of 15 and false discovery rate (FDR) of peptides was set up to less than 1%. The accurate quantification of proteins by ^15^N-metabolic labeling and Mascot Distiller quantitation was performed according to the protocol reported previously^25^.

The inter-crosslinked peptides were analyzed by Batch-Tag Web database search (ProteinProspector, University of California, San Francisco)^15^. The MGF data were searched against the in-house database containing the sequences of 12 subunits of the Pol V complex and 17 potential interacting proteins identified by relative quantification of proteins with ^15^N-metabolic labeling. The BS^3^ cross-linked peptides matching decoy protein sequences and less than 6 amino acids were excluded, and only those with the score difference greater than 5 and the ion score greater than 30 were considered.

### Construction of the functional protein NRPE1 interaction network

The putative interacting proteins were submitted to the STRING database (http://string-db.org/) to generate the functional protein interaction network of NRPE1. Predicted protein interaction network from the Retrieval of Interacting Genes/Proteins (STRING) databases were loaded into the Cytoscape program (v.3.4) (http://www.cytoscape.org/) to visualize protein interaction pathways. Structure modeling of NRPE1 and NRPE2 was performed using Phyre2 server (http://www.sbg.bio.ic.ac.uk/phyre2/).

The mass spectrometry proteomics data have been deposited to the ProteomeXchange Consortium *via* the PRIDE partner repository with the dataset identifier PXD005422^26^.

## Additional Information

**How to cite this article:** Qin, G. *et al*. Methylated-antibody affinity purification to improve proteomic identification of plant RNA polymerase Pol V complex and the interacting proteins. *Sci. Rep.* **7**, 42943; doi: 10.1038/srep42943 (2017).

**Publisher's note:** Springer Nature remains neutral with regard to jurisdictional claims in published maps and institutional affiliations.

## Supplementary Material

Supplementary Information

## Figures and Tables

**Table 1:** Identification of post-translational modifications of NRPE1 by LC MS/MS analyses and database search.

m/z	z	Mr (expt)	Mr (calc)	ppm	score	peptide sequence	modification
901.0477	3	2700.1212	2700.1129	3.2	51	^1293^FED**S**ADFQNLHDEGKPSGANWEK^1315^	_P_S^1296^
529.2221	2	1056.4295	1056.4277	1.7	39	^1372^SD**S**GGAWGIK^1381^	_P_S^1374^
948.3885	2	1894.7624	1894.7622	1.1	31	^1384^DADADT**T**PNWETSPAPK^1400^	_P_T^1390^
948.3856	2	1894.7566	1894.7622	−2.9	67	^1384^DADADTTPNWET**S**PAPK^1400^	_P_S^1396^
964.4177	2	1926.8210	1926.8183	1.5	97	^1568^KN**S**ETELGPAAMGNWDK^1584^	_P_S^1570^
1202.5381	2	2403.0606	2403.0553	2.2	47	^1729^LDSFTSEEQELL**S**DVEPVMR^1748^	_P_S^1741^
861.8885	2	1721.7614	1721.7605	0.6	61	^1453^**K**NSETESDAAAWGSR^1467^	_Ub_K^1453^
966.9541	2	1931.8936	1931.8861	2.9	26	^1517^**K**NIETDSEPAAWGSQGK^1533^	_Ub_K^1517^

_*p*_S/T: phosphorylated Ser/Thr; _Ub_K: ubiquitinated Lys.
