# Supplementary material for: Methylated-antibody affinity purification to improve proteomic identification of plant RNA polymerase Pol V complex and the interacting proteins
Source: Sci Rep. 2017 Feb 22;7:42943. doi: 10.1038/srep42943 (PMC5320500; doi:10.1038/srep42943)
Supplement: Supplementary Information [file srep42943-s1.pdf]

Supplementary Information for

Methylated-antibody affinity purification to improve proteomic identification of plant RNA polymerase Pol V complex and the interacting proteins

Guochen Qin[1], Jun Ma[1], Xiaomei Chen[1], Zhaoqing Chu[2*], Yi-Min She[1,3]*

[1]Shanghai Center for Plant Stress Biology, [2]Shanghai Chenshan Plant Science Research Center and Shanghai Chenshan Botanic Garden, Shanghai Institutes for Biological Sciences, Chinese Academy of Sciences, 3888 Chenhua Road, Shanghai 201602, P. R. China. [3]Centre for Biologics Evaluation, Biologics and Genetic Therapies Directorate, Health Canada, Ottawa, Ontario, K1A 0K9, Canada. *Correspondence and requests for materials should be addressed to Y.M.S. (e-mail: yiminshe@gmail.com) or Z.C. (e-mail: zqchu@sibs.ac.cn)

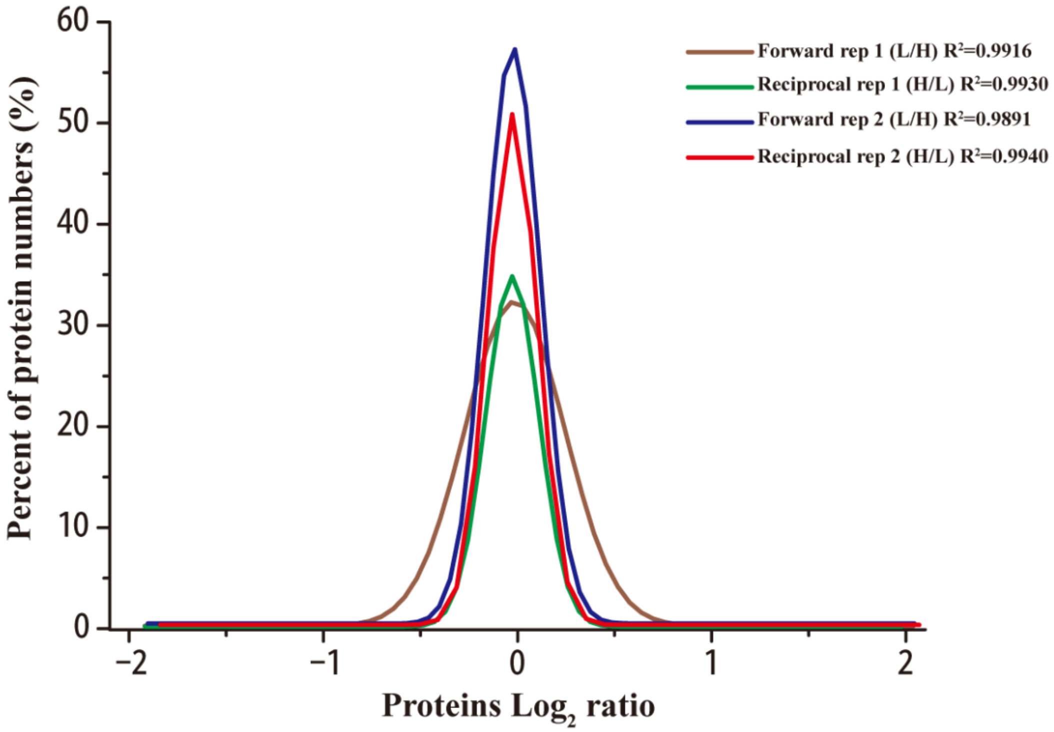

Supplemental Figure S1. The distributions of protein log_2 ratios derived from the dimethylated antibody immunoprecipitation, enzymatic digestion and quantitative analyses of $^{14}N/^{15}N$-metabolic labeling *Arabidopisis* between wild-type and NRPE1-FLAG transgenic plants. The log_2-fold change in each experiment obtained from to total number of quantified proteins follows a Gaussian distribution with the coefficient of determination (R^2) to be greater than 0.98 in both "Forward" and "Reciprocal" replicates. Mascot Distiller with the aid of data manual inspection provided the accurate quantitative protein ratios of ^{14}N to ^{15}N labeling peaks.

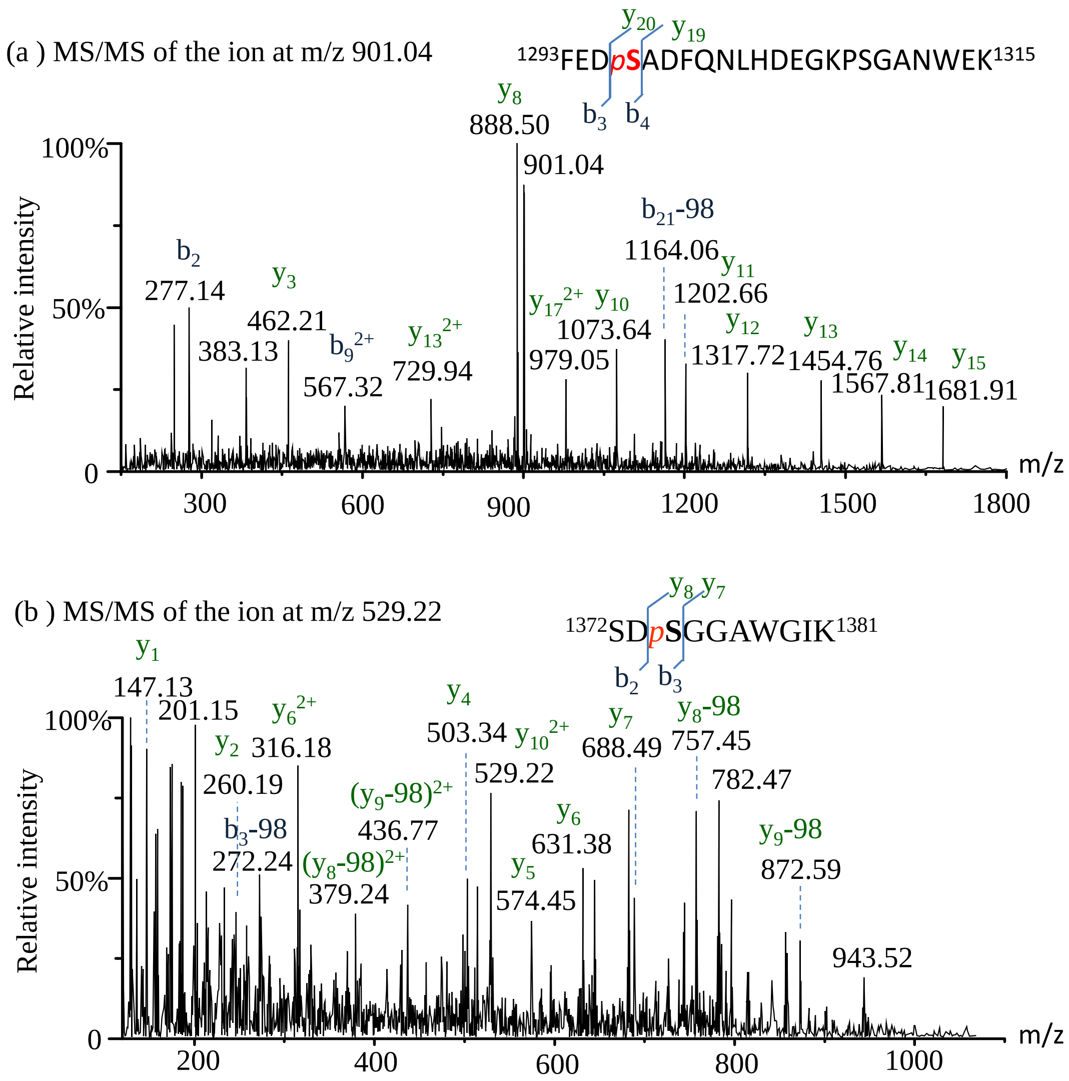

Supplemental Figure S2. Identification of phosphorylation sites of NRPE1. (a) MS/MS spectrum of the triply charged ion at m/z 901.0477 displayed a series of the fragments up to the N-terminal b_2 ion and C-terminal y_{17} ion without mass change, and identified the phosphorylation site at Ser1296 of peptide 1293-1315. (b) MS/MS spectrum of the doubly charged ion at m/z 529.22 showed the unchanged masses at the C-terminal fragment ions up to y_7, and the loss of 98 Da (phosphate group) starting at y_8 ion defined the phosphorylation site at Ser1374 of peptide 1372-1381.

(c) MS/MS of the ion at m/z 948.39

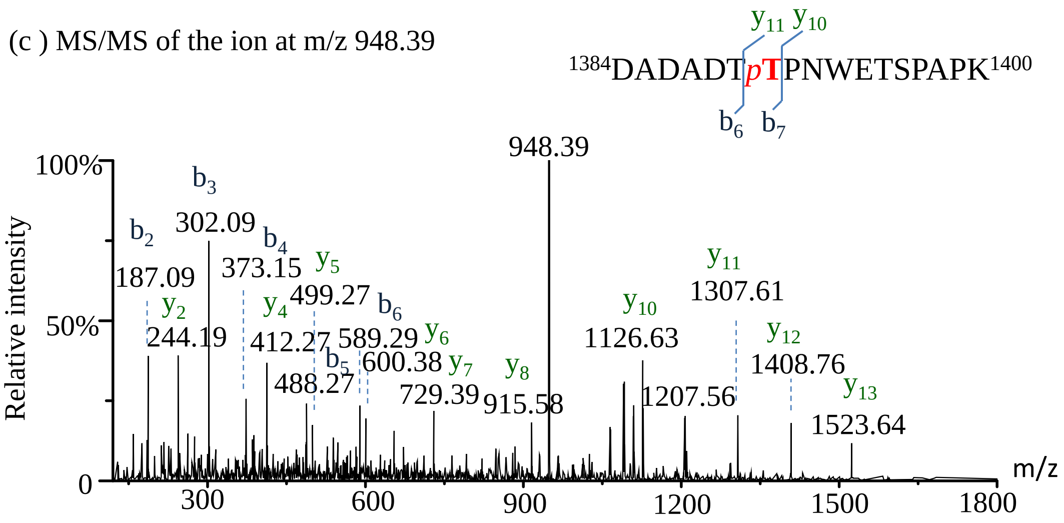

(d) MS/MS of the ion at m/z 948.38

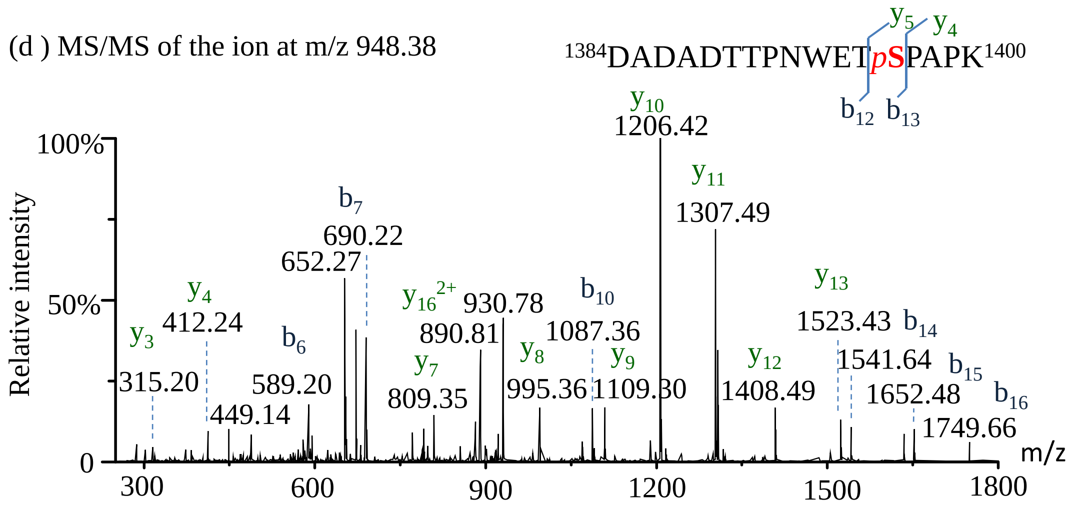

Supplemental Figure S2. Identification of phosphorylation sites of NRPE1. (c) MS/MS spectrum of the doubly charged ion at m/z 948.3885 displayed a series of fragments up to N-terminal b_6 ion and C-terminal y_{10} ion without mass change, and 181 Da mass difference of the phosphorylated Thr1390 between y_{10} and y_{11} ions of peptide 1384-1400. (d) MS/MS spectrum of the doubly charged ion at m/z 948.3856 showed the unchanged masses at the N-terminal b_{10} ion and the C-terminal fragment ions up to y_4, and the mass increase of 80 Da (phosphate group) starting at y_7 ion and b_{10} defined the phosphorylation site at Ser1396 of peptide 1384-1400.

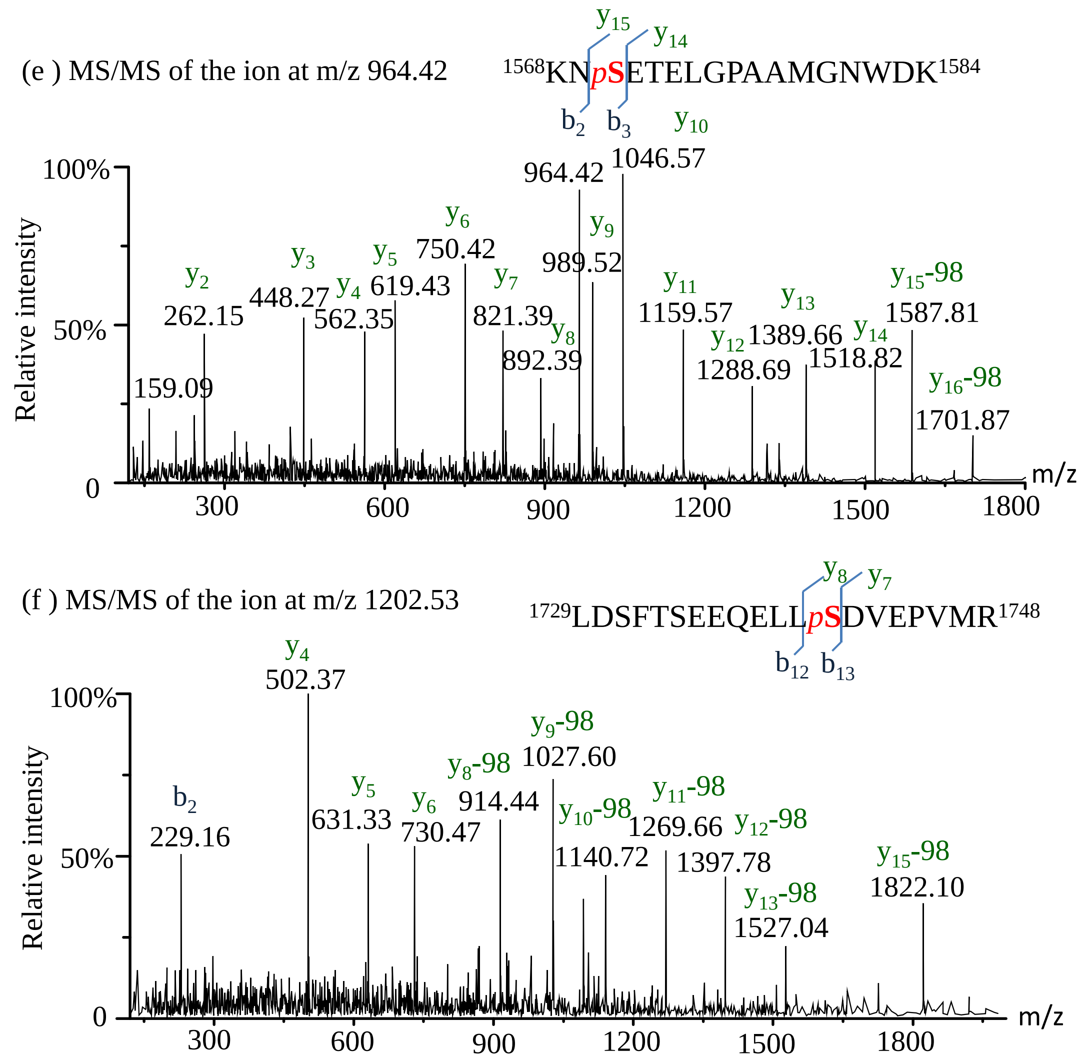

Supplemental Figure S2. Identification of phosphorylation sites of NRPE1. (e) MS/MS spectrum of the ion at m/z 964.4177 displayed a series of the fragments up to the C-terminal y_{14} ion without mass change, and the loss of 98 Da (phosphate group) starting at y_{15} ion defined the phosphorylation site at Ser1570 of peptide 1568-1584. (f) MS/MS spectrum of the ion at m/z 1202.5380 showed the unchanged masses at the C-terminal fragment ions up to y_6, and the loss of 98 Da (phosphate group) starting at y_8 ion defined the phosphorylation site at Ser1741 of peptide 1729-1748.

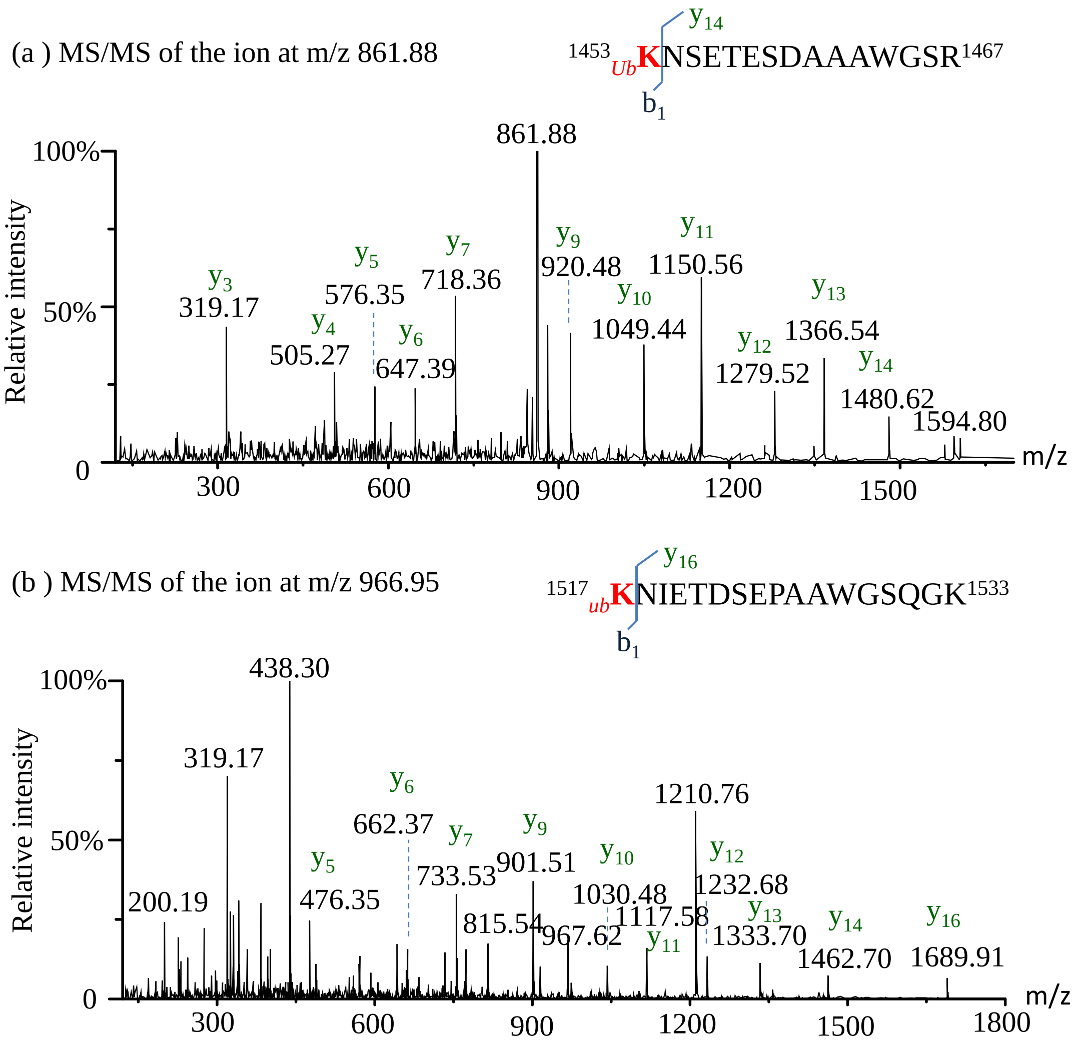

Supplemental Figure S3. Identification of ubiquitination sites of NRPE1. (a) MS/MS spectrum of the doubly charged ion at m/z 861.8880 displayed a series of the fragments up to the C-terminal y_{14} ion without mass change, and the increased mass of 114 Da (diglycine group) ending at y_{15} ion defined the ubiquitination site at Lys1453 of peptide 1453-1467. (b) MS/MS spectrum of the doubly charged ion at m/z 966.9541 showed the unchanged masses at the C-terminal fragment ions up to y_{16}, and the increased mass of 114 Da (diglycine modification) ending at y_{17} ion defined the ubiquitination site at Lys1517 of peptide 1517-1533.

Supplemental Table S1. Relative contribution of the peptide-spectrum-match (PSM) numbers of antibodies and Pol V complex subunits by immunoaffinity purification, on-bead digestion and LC MS/MS analyses

Protein	Non-methylated antibody - Control 1 (%)				Non-methylated antibody - Control 2 (%)				Dimethylated antibody (%)			
	Rep.1	Rep.2	Rep.3	Mean±SD	Rep.1	Rep.2	Rep.3	Mean±SD	Rep.1	Rep.2	Rep.3	Mean±SD
Antibody chains												
Ab light	35.89	34.52	37.90	36.10±1.70	24.78	22.71	28.97	25.49±3.19	13.21	7.08	10.61	10.30±3.08
Ab heavy	13.97	16.53	13.15	14.55±1.76	9.99	7.65	10.60	9.41±1.56	1.77	2.10	2.22	2.03±0.23
Subtotal	49.85	51.05	51.05	50.65±0.69	34.77	30.36	39.57	34.90±4.61	14.98	9.18	12.83	12.33±2.93
Pol V subunits												
NRPE1	30.29	28.43	27.15	28.62±1.58	37.10	38.53	35.00	36.88±1.78	42.77	46.35	46.26	45.13±2.04
NRPE2	5.60	4.48	5.59	5.22±0.64	7.36	7.87	6.38	7.20±0.76	12.07	11.28	9.19	10.85±1.49
NRPE3a	3.65	4.48	3.66	3.93±0.48	6.05	7.19	6.72	6.65±0.57	12.17	13.05	12.22	12.48±0.49
NRPE3b	1.36	1.82	1.80	1.66±0.26	2.55	3.63	3.05	3.08±0.54	4.27	4.65	5.15	4.69±0.44
NRPE4	0.53	0.77	0.78	0.69±0.14	1.31	0.98	1.18	1.16±0.16	1.35	1.55	1.31	1.40±0.13
NRPE5	2.30	1.75	1.92	1.99±0.28	1.90	1.89	1.80	1.86±0.05	3.02	2.65	1.92	2.53±0.56
NRPE6a	0.59	0.63	0.72	0.65±0.07	0.80	1.06	0.49	0.78±0.29	0.62	1.44	1.41	1.16±0.46
NRPE6b	0.47	0.49	0.36	0.44±0.07	0.73	0.53	0.14	0.47±0.30	0.62	0.88	0.81	0.77±0.13
NRPE7	1.30	1.61	1.62	1.51±0.18	2.04	1.97	1.18	1.73±0.48	2.60	2.10	2.42	2.38±0.25
NRPE8a	0.65	0.77	0.96	0.79±0.16	1.17	0.61	0.62	0.80±0.32	0.62	0.66	0.71	0.67±0.04
NRPE8b	0.47	0.77	1.08	0.77±0.30	1.31	1.21	0.90	1.14±0.21	0.62	1.11	1.31	1.01±0.35
NRPE9a	0.24	0.28	0.18	0.23±0.05	0.15	0.30	0.35	0.27±0.11	0.42	0.33	0.30	0.35±0.06
NRPE9b	0.71	0.91	0.72	0.78±0.11	0.29	0.91	0.97	0.72±0.38	0.52	1.00	1.11	0.88±0.31
NRPE10	0.47	0.56	0.36	0.46±0.10	0.80	0.61	0.35	0.58±0.23	1.25	0.44	0.51	0.73±0.45
NRPE11	1.41	0.98	1.86	1.42±0.44	1.46	2.12	1.11	1.56±0.51	1.87	3.10	2.32	2.43±0.62
NRPE12	0.12	0.21	0.18	0.17±0.05	0.22	0.23	0.21	0.22±0.01	0.21	0.22	0.20	0.21±0.01
Pol V	50.15	48.95	48.95	49.35±0.69	65.23	69.64	60.43	65.10±4.61	85.02	90.82	87.17	87.67±2.93

The data were collected from three sets of biologically repeated experiments at Mean ± SD (standard deviation). "Control 1": the proteins were immunoprecipitated by non-methylated anti-FLAG antibodies and digested on-beads by trypsin; "Control 2": the proteins were immunoprecipitated by non-methylated anti-FLAG antibodies and digested on-beads by endoproteinase Lys-C, and the eluted peptides were further cleaved by trypsin; "Dimethylated antibody": the proteins were immunoprecipitated by dimethylated antibodies and digested on-beads with endoproteinase Lys-C, and then the peptides were eluted and subjected to additional digestion by trypsin.

Supplemental Table S2. Identification of the inter-cross-linked peptides between Pol V complex subunits and the interacting proteins

m/z	z	ppm	score	Peptide 1 sequence	Peptide 2 sequence	Protein 1 (gene #)	Protein 2 (gene #)
Cross-linked peptides among the protein subunits of Pol *V* complex							
786.809	5	-9.9	41.5	^{386}DGS**K**^{389}GHTELKPGQ(De)VVHR^{402}	**K**^{822}MDELVQ(De)FGKTHSKIGK^{838}	NRPE1(AT2G40030)	NRPE2(AT3G23780)
1500.166	3	-5.9	41.2	^{532}SS**K**^{534}SGPAWTVFQILQLAFPER^{552}	^{132}I**K**^{133}VNVQVEVFK(Xlink:DSS2)NTVVK^{147}	NRPE1(AT2G40030)	NRPE2(AT3G23780)
870.757	3	0.5	32.2	^{716}TLVEDMAIFC(Cam)**K**^{726}R^{727}	^{612}D**K**^{613}DDNEVR^{619}	NRPE1(AT2G40030)	NRPE2(AT3G23780)
954.495	5	-8.7	41.8	^{727}R**K**^{728}YGRISSSGDFGIVK^{742}	^{455}AFSTGAWSHPFR**K**^{467}MERVSGVVAN(De)LGR^{480}	NRPE1(AT2G40030)	NRPE2(AT3G23780)
1153.606	4	9.4	36.5	^{1839}AEEFID**K**^{1845}YFTKPRPSGN(De)RDR^{1858}	^{393}DSFRN(De)**K**^{398}RIELAGELLER^{409}	NRPE1(AT2G40030)	NRPE2(AT3G23780)
567.889	7	-14.0	37.4	^{1846}YFT**K**^{1849}PRPSGNRDR^{1858}	^{162}KILDVK**K**^{168}Q(De)DILIGSIPVMVK^{181}	NRPE1(AT2G40030)	NRPE2(AT3G23780)
1310.719	3	-12.0	45.1	^{245}K(Xlink:DSS2)VIAI**K**^{250}SSRSGETNFESHK^{263}	^{167}AIAR**K**^{171}GIGK(Xlink:DSS2)DHAK^{279}	NRPE1(AT2G40030)	NRPE3a(AT2G15430)
568.0314	7	-5.4	36.8	^{643}SNSAIT**K**^{649}LVQQ(De)TGFLGLQLSDKK^{665}	^{85}V**K**^{86}IVFFGTSMVK^{96}	NRPE1(AT2G40030)	NRPE5(AT3G57080)
882.6284	5	-15	39.5	^{143}NTVV**K**^{147}SDK(Xlink:DSS1)FKTGQDN(De)YVEK^{1461}	^{1468}D**K**^{1469}NNSDVGSGAGVLGPWNK^{1486}	NRPE2(AT3G23780)	NRPE1(AT2G40030)
1225.637	3	7.9	38.1	**K**^{388}C(Cam)EN(De)RDSFR^{396}	^{668}EVAANFML**K**^{676}SYSIRNLIDIK^{687}	NRPE2(AT3G23780)	NRPE1(AT2G40030)
745.040	3	-0.7	30.0	^{1070}FGGI**K**^{1074}FGEMER^{1080}	**K**^{318}GSGFSSR^{325}	NRPE2(AT3G23780)	NRPE1(AT2G40030)
930.830	3	-19.0	52.4	^{108}LSS**K**^{111}C(Cam)VTDQTLDVTSR^{123}	**K**^{162}ILDVKK^{168}	NRPE3a(AT2G15430)	NRPE2(AT3G23780)
737.398	4	-13.0	44.8	^{108}LSS**K**^{111}C(Cam)VTDQTLDVTSR^{123}	^{162}KILDV**K**^{167}K^{168}	NRPE3a(AT2G15430)	NRPE2(AT3G23780)
1099.223	3	4.4	37.8	^{612}D**K**^{613}DDNEVRIFTDAGR^{626}	^{15}S**K**^{16}DGGKDGSSTKLK^{28}	NRPE2(AT3G23780)	NRPE4(AT4G15950)
1291.720	3	-3.3	45.7	^{11}SSLK(Xlink:DSS2)S**K**^{16}DGGK(Xlink:DSS2)DGSSTK^{26}	^{132}I**K**^{133}VNVQVEVFKNTVVK^{147}	NRPE4(AT4G15950)	NRPE2(AT3G23780)
604.903	7	-12.0	43.9	**K**^{188}DAIVRYYGLEKGQVVK^{204}	^{903}LN(De)**K**^{905}VSLKDTAVEFLVEYR^{920}	NRPE5(AT3G57080)	NRPE1(AT2G40030)
1305.047	3	-1.8	36.5	^{50}VETEPVQRPR**K**^{60}TSKFMTK^{67}	^{390}GHTEL**K**^{395}PGQVVHRR^{403}	NRPE6a(AT5G51940)	NRPE1(AT2G40030)
Cross-linked peptides between interacting proteins and NRPE1 in Pol *V* complex							
679.621	7	-1.5	35.5	**K**^{1517}NIETDSEPAAWGSQGK(Xlink:DSS2)K^{1534}	^{478}L**K**^{479}MGC(Cam)GSETFPRNGRWNFNNK^{498}	NRPE1(AT2G40030)	AGO4(AT2G27040)
792.723	6	-4.3	34.0	**K**^{1568}NSETELGPAAMGNWDK(Xlink:DSS1)K(Xlink:DSS1)K^{1586}	^{425}DN(De)IC(Cam)**K**^{429}ADIEEVTSLYLDAK^{443}	NRPE1(AT2G40030)	RIN1(AT5G22330)
1236.365	4	-3.1	36.4	**K**^{414}GK(Xlink:DSS2)VVEVEDIQR^{425}	^{1424}SWDK(Xlink:DSS2)**K**^{1428}NWGTESAPAAWG STDAAVWGSSDK^{1452}	RuvB-like helicase (AT5G67630)	NRPE1(AT2G40030)

The BS^3-crosslinked peptides were analyzed by MS-Bridge (Protein Prospector, University of California at San Francisco). Cam, Carbamidomethylation of cysteine; De, Deamidation; Xlink:DSS1 and Xlink:DSS2, dead-end modification with the BS^3 cross-linker (where one end reacts with a primary amine in the peptide but the other is hydrolyzed).

Supplemental Table S3. Quantification of potential interacting proteins with NRPE1 by ^{15}N-metabolic labeling and dimethylated antibody affinity purification

Gene accession	Protein	Forward L/H (Mean±SD)	Reciprocal H/L (Mean±SD)	Forward L/H (Mean±SD)	Reciprocal H/L (Mean±SD)
AT2G27040	AGO4	2.55±0.35	3.23±0.12	2.37±0.49	3.37±0.21
AT2G30280	RDM4	1.95±0.21	1.75±0.05	2.12±0.05	1.51±0.13
AT5G22330	RIN1	4.16±0.66	7.35±0.09	7.57±0.17	13.61±0.13
AT5G67630	RuvB-like helicase	3.32±0.14	11.33±1.38	7.77±0.14	14.53±0.46
AT3G60830	ARP7	5.25±0.06	10.54±1.04	5.18±0.47	8.71±0.32
AT2G40660	OB-fold-like protein	1.65±0.35	1.85±0.22	2.55±0.38	2.28±0.11
AT5G02500	HSP70-1	1.61±0.31	3.66±0.11	2.29±0.26	7.07±0.11
AT3G09440	HSP70-3	1.77±0.28	4.13±0.19	2.26±0.13	5.74±0.28
AT5G56030	HSP81-2	8.06±0.19	13.01±0.73	5.31±0.11	4.39±0.09
AT3G44110	J3	2.50±0.30	1.96±0.08	2.26±0.19	2.21±0.37
AT2G20140	RPT2B	1.88±0.22	2.10±0.13	2.46±0.04	2.44±0.07
AT2G21660	GRP7	1.72±0.41	1.56±0.06	2.16±0.26	2.12±0.16
AT3G08590	IPGAM2	2.53±0.22	3.46±0.24	2.55±0.25	2.66±0.18
AT3G16420	PBP1	2.30±0.13	3.07±0.15	3.11±0.35	4.52±0.35
AT1G13930	unknown protein	1.97±0.13	2.46±0.40	2.25±0.33	1.80±0.26
AT3G44690	unknown protein	6.49±0.27	2.65±0.49	4.57±0.24	6.71±0.16

Experiments were conducted with two biological duplicates. SD, standard deviation.
